# Integrating machine learning and multi-omics analysis to reveal nucleotide metabolism-related immune genes and their functional validation in ischemic stroke

**DOI:** 10.3389/fimmu.2025.1561544

**Published:** 2025-03-26

**Authors:** Tianzhi Li, Xiaojia Kang, Sijie Zhang, Yihan Wang, Jinshan He, Hongyan Li, Chen Shao, Jingsong Kang

**Affiliations:** Department of Pathophysiology, Key Laboratory of Pathobiology, Ministry of Education, College of Basical Medical Sciences, Jilin University, Changchun, China

**Keywords:** ischemic stroke, nucleotide metabolism, molecular docking, bioinformatics analysis, machine learning

## Abstract

**Background:**

Ischemic stroke (IS) is a major global cause of death and disability, linked to nucleotide metabolism imbalances. This study aimed to identify nucleotide metabolism-related genes associated with IS and explore their roles in disease mechanisms for new diagnostic and therapeutic strategies.

**Methods:**

IS gene expression data were sourced from the GEO database. Differential expression analysis and weighted gene co-expression network analysis (WGCNA) were conducted in R, intersecting results with nucleotide metabolism-related genes. Functional enrichment and connectivity map (cMAP) analyses identified key genes and potential therapeutic agents. Core immune-related genes were determined using LASSO regression, SVM-RFE, and Random Forest algorithms. Immune cell infiltration levels and correlations were analyzed via CIBERSORT. Single-cell RNA sequencing (scRNA-seq) data and molecular docking assessed gene expression, localization, and gene-drug binding. *In vivo* experiments validated core gene expression.

**Results:**

Thirty-three candidate genes were identified, mainly involved in immune and inflammatory responses. *CFL1, HMCES*, and *GIMAP1* emerged as key immune-related genes, linked to immune cell infiltration and showing high diagnostic potential. cMAP analysis indicated these genes as drug targets. scRNA-seq clarified their expression and localization, and molecular docking confirmed strong drug binding. *In vivo* experiments validated their significant expression in IS.

**Conclusion:**

This study underscores the role of nucleotide metabolism in IS, identifying *CFL1, HMCES*, and *GIMAP1* as potential biomarkers and therapeutic targets, providing insights for IS diagnosis and therapy development.

## 1 Introduction

Ischemic stroke (IS) is a disease of the central nervous system with a complex pathogenesis, and morbidity and mortality of stroke have been increasing in recent years, representing an escalating public health challenge (1). Pathologically, ischemic stroke is characterised by disruption of the local blood supply to the brain tissue leading to tissue ischemia and hypoxia, involving a variety of molecular mechanisms and cytological changes including oxidative stress, inflammatory cascades, activation of apoptotic pathways, and dysfunction of the blood-brain barrier. Nucleotide metabolism plays a key role in this process (2). Nucleotides are not only important components of cellular energy metabolism and signalling, but are also involved in cell growth, division and repair. Disturbances in nucleotide metabolism during ischaemia can lead to a decrease in ATP synthesis, which in turn affects cellular energy supply and viability (3). In addition, the breakdown products of nucleotides, such as adenosine, may further affect the extent of brain tissue damage and the ability to recover by regulating the inflammatory response and the apoptotic pathway after ischaemia (4). Therefore, an in-depth study of the pathogenesis of ischemic stroke and its regulatory network, especially the role of nucleotide metabolism, is of great importance for the development of novel therapeutic strategies and the improvement of patient prognosis.

Nucleotide metabolism is a complex process of intracellular nucleotide synthesis, degradation and recycling that is critical for cellular energy metabolism and signalling (5). Numerous studies have shown that nucleotide metabolism plays a key role in the onset and development of ischaemic brain injury (6). First, an imbalance in the synthetic pathway leads to a disruption of energy metabolism in cerebral ischaemia, resulting in the rapid breakdown of ATP to ADP and AMP, which are further converted to metabolites such as adenosine, inosine and hypoxanthine (7). Adenosine is an important neuromodulator that regulates neuronal excitability, vasodilation and inflammatory responses by binding to adenosine receptors (A1, A2A, A2B and A3) (8). Secondly, an imbalance in the catabolic pathway allows the accumulation of metabolites (e.g. uric acid) from nucleotide degradation during acute ischaemia, leading to oxidative stress and cellular damage that further exacerbates brain injury (9).In addition, degradation products of purine nucleotides (e.g. xanthine) are oxidised by xanthine oxidase (XO) during ischaemia-reperfusion, generating large amounts of reactive oxygen radicals and exacerbating oxidative stress injury (10). Finally, imbalances in energy and adenosine metabolism may affect DNA repair and cellular energy homeostasis, thereby compromising neuronal survival and functional recovery. Although studies have demonstrated the potential importance of nucleotide metabolism in ischemic stroke, studies on effective diagnostic biomarkers of nucleotide metabolism in ischemic stroke are still lacking and need to be further investigated.

In this study, we used a bioinformatics approach to comprehensively analyse nucleotide metabolism genes associated with ischemic stroke (IS) and their biological functions, and to validate them *in vivo*. First, datasets related to IS and nucleotide metabolism were retrieved from the GEO database and GeneCards, and samples were classified using differential expression analysis and weighted gene co-expression network (WGCNA) construction to reveal core IS genes related to nucleotide metabolism and their potential mechanisms. Multiple machine learning algorithms were then applied to screen to obtain three key genes, and the accuracy and generalisability of the constructed models were assessed by receiver operating characteristic curve (ROC) analysis. Based on single-cell sequencing data, we further analysed the expression distribution of these key genes in different cell types. In addition, we identified compounds with potential therapeutic effects on IS and evaluated their binding ability using molecular docking techniques. Finally, a rat cerebral ischaemia model was established to validate the expression differences of the key genes in normal and IS samples by qPCR to explore their potential as diagnostic and therapeutic targets for IS.

## 2 Methods

### 2.1 Data acquisition and preprocessing

**Figure 1:**
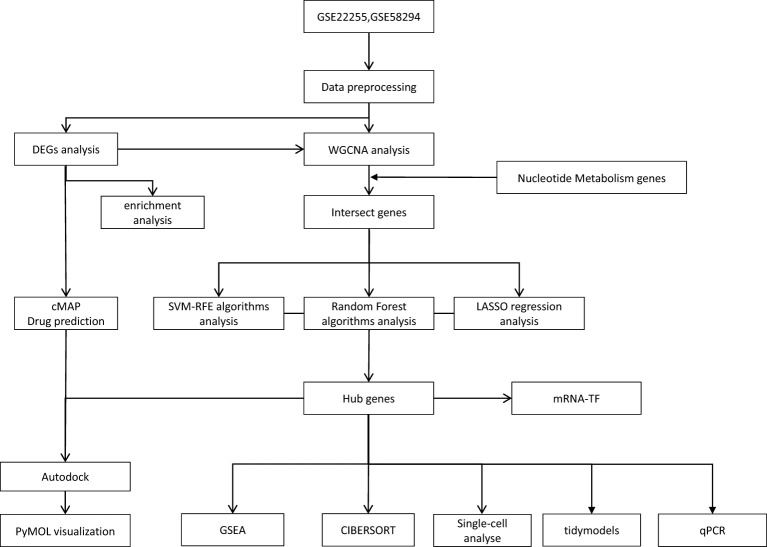
Study workflow.

In this study, we analysed samples from IS and normal controls to investigate the key genes involved in nucleotide metabolism in IS. We downloaded two microarray datasets from the GEO database: the GSE22255 and the GSE58294, both belonging to the GPL570 platform (HG-U133_Plus_2) Affymetrix Human Genome U133 Plus 2.0], and a single-cell RNA sequencing (scRNA-seq) dataset (GSE174574). The NM (Nucleotide Metabolism) gene set for nucleotide metabolism was obtained from the Genecards website, with the criterion of a relevance score ≥1 to ensure biological relevance. Specifically, GSE22255 and GSE58294 were used as screening sets and GSE174574 as validation set. After the steps of data pre-processing, batch effect correction using the combat function, and expression value aggregation, we obtained the final gene expression matrix. The details are shown in **Supplementary Table 1**, and the overall study flow is shown in **Figure 1**.

### 2.2 Identification of differentially expressed genes

To identify differentially expressed genes (DEGs) between normal and IS samples, we used the limma package (3.46.0) (11) in R to perform differential expression analysis. Screening criteria were set as p-value < 0.05 and |log2(fold change)| > 0.5. Heatmaps and volcano plots were generated for visualisation using the ggplot2 package (1.0.1) to further demonstrate the expression characteristics of DEGs. We then performed an intersection analysis of the screened DEGs with nucleotide metabolism genes using the Venn diagram package (1.7.3) to identify nucleotide genes that were differentially expressed in the pathogenesis of IS.

### 2.3 Weighted gene co-expression network analysis

The data from GSE22255 and GSE58294 were merged and batch processed. The weighted gene co-expression network analysis (WGCNA) software package (1.73) (12) was used to evaluate the trait-related modules. Topological overlap matrices were constructed based on expression curves. A soft threshold of 6 and a minimum module size of 30 were used to filter the core modules, and a height constraint of 0.25 was used as a guide for module combinations. Modules were then tested using the Pearson correlation test with a significance threshold of *P* < 0.05. Candidate genes were also found by taking the intersection of differentially expressed nucleotide genes with genes from the WGCNA core module.

### 2.4 Enrichment analysis

To identify biological processes, metabolic pathways with candidate genes, we performed GO, KEGG pathway and disease enrichment analyses using the enrichGO and enrichKEGG functions in clusterProfiler package (4.12.6) (13).

### 2.5 Machine learning model building

In order to rank the importance of genes and identify potential biomarkers, we employed three commonly used machine learning models for screening and classification of feature genes. In all the algorithms, the merged IS dataset was randomly divided into a training set and a test set in the ratio of 7:3, where the training set was used for model training and parameter optimisation, and the test set was used to evaluate the generalisation ability of the models. These models include the Support Vector Machine Recursive Feature Elimination (SVM-RFE) method using the e1071 package (version 1.7-16) (14), the Random Forest (RF) algorithm using the randomForest R package (version 4.7-1.2) (15), and the Lasso Regression (LASSO) algorithm using the glmnet package (version 4.1-8) (16). Specifically, LASSO is a linear model for feature selection and regression analysis via L1 regularisation, which can effectively handle high-dimensional data and reduce overfitting, thus improving model stability. In this study, 10-fold cross-validation was used to select the best lambda values corresponding to the key genes. Random Forest is an integrated learning algorithm that improves overall prediction accuracy and robustness by training multiple decision trees on a random subset of data and integrating their predictions, while SVM-RFE ranks the importance of features by evaluating their contribution to the classifier performance. Finally, we performed a Venn diagram analysis of the feature genes screened by these three models, and selected the genes that consistently appeared as key genes to lay the foundation for subsequent biomarker studies.

### 2.6 Assessment of pivotal genes

In order to evaluate the diagnostic efficacy of the screened signature genes, we first constructed a diagnostic column-line graph model using the rms package. In the model, ‘Points’ represent the scores of each corresponding factor, and the corresponding calibration curves were plotted to evaluate the predictive accuracy of the model. Secondly, the pROC package (v1.18.0) (17) was used to construct Receiver Operating Characteristic (ROC) curves and calculate the Area Under Curve (AUC). The AUC was calculated to evaluate the classification performance of the characterised genes under different sensitivity and specificity conditions, and the closer the AUC was to 1, the better the diagnostic performance. Meanwhile, to visualise the expression differences of the feature genes between different sample groups, the ggplot2 package (v3.4.0) was used to construct box plots to show the expression distribution of the feature genes.

### 2.7 Hub gene enrichment and immune infiltration

Single-gene GSEA was used to explore the relationship between hub genes and disease states and their regulatory mechanisms (18). CIBERSORT is an inverse convolutional algorithm capable of transforming a normalised gene expression matrix into the composition of infiltrating immune cells. We compared the infiltration of 22 immune cells in normal and ischemic stroke (IS) samples in the combined IS datase. LM22 was used as the reference expression signature and 1000 permutations were performed to improve the accuracy of predicting immune cell composition. CIBERSORT output was defined as p < 0.05 and eligible samples were then selected for further analysis. The 22 types of infiltrating immune cells included B cells (naive B cells and memory B cells), T cells (CD8 T cells, naive CD4 T cells, memory resting CD4 T cells, memory activated CD4 T cells, follicular helper T cells, regulatory T cells), and γδ T cells), NK cells (resting and activated NK cells), monocytes, macrophages (M0, M1 and M2 type macrophages), dendritic cells (resting and activated dendritic cells), mast cells (resting and activated mast cells), eosinophils and neutrophils. The composition of all 22 immune cell types assessed was summed to 1 in each sample (19).

### 2.8 Diagnostic model construction and mRNA-TF network analysis

In order to comprehensively evaluate the prediction performance of key genes and their molecular regulatory mechanisms, the study adopts a multi-dimensional analysis strategy that integrates machine learning modelling and transcriptional regulatory network analysis. In terms of machine learning model construction, model training, evaluation and comparison were achieved based on Tidymodels framework (v1.2.0) (20). The generalisation ability and prediction stability of the model were ensured by 10-fold cross-validation. Meanwhile, to deeply resolve the transcriptional regulatory networks of key genes, TF-mRNA interaction maps were constructed using the NetworkAnalyst website(https://www.networkanalyst.ca/) (21) and imported into Cytoscape software for visualisation. By combining the machine learning prediction model with the transcriptional regulatory network analysis, we not only verified the diagnostic value of the key genes, but also elucidated their potential molecular regulatory mechanisms, laying a theoretical foundation for the subsequent functional validation experiments and clinical translational applications.

### 2.9 Analysis of single cell data and intercellular communication

We obtained the single-cell RNA sequencing dataset GSE174574 from the GEO database and performed systematic quality control and downstream analysis of the dataset using Seurat (version 5.0.1) (22). First, cells with gene expression below 200 or above 3000 were excluded, as well as genes detected in fewer than 3 cells. In addition, cells with more than 20% unique molecular identifier (UMI) counts of mitochondrial origin were filtered out. The data set was then log-normalised and the top 2000 highly variable genes (HVGs) were selected for typical correlation analysis. The ScaleData function was then used to normalise the data and Principal Component Analysis (PCA) was performed. Based on the PCA results, cell clustering was performed using the first 10 principal components with a resolution parameter of 0.5. t-SNE and UMAP visualisation maps were also generated to show the distribution and characteristics of the cell population. Cell classification was also performed using SingleR and CellMarker 2.0 (23) and the corresponding visualisation analysis was performed. Subsequently, cell-to-cell communication analysis was performed using the CellChat package (version 1.6.1) to provide a comprehensive understanding of the dynamics of the cellular microenvironment during the pathogenesis of ischemic stroke (IS).

### 2.10 Expression level analysis in normal human tissues

Expression of key genes in human tissues is analysed using the harmonizome database (https://maayanlab.cloud/Harmonizome/) (24).

### 2.11 CMAP analysis

Drug prediction analyses have been performed to gain a deeper understanding of drug mechanisms and to discover new therapeutic compounds. The Connection Mapping (CMAP) database contains 6100 instances of 1309 small molecule drugs, each accompanied by a gene expression profile for a specific drug and its corresponding treatment. In this study, we used gene expression profiles to predict potential small molecule compounds for the treatment of ischemic stroke (IS) based on the CMAP database. First, we uploaded differentially expressed genes (DEGs) into the CMAP database to predict possible therapeutic small molecule drugs. The CMAP scores range from -100 to 100, with a negative value indicating that the gene expression profile of the compound is negatively correlated with the disease state, suggesting that it may have therapeutic potential. This analysis provides strong support for the screening of candidate compounds that merit further validation, thus laying the groundwork for future therapeutic studies.

### 2.12 Protein-ligand interaction analysis

To further validate the candidate compounds predicted by the CMAP analysis, we performed protein-ligand molecular docking experiments. First, the 3D structures of the core target proteins were obtained from the UniProt database, and the structure files of the CMAP-predicted small molecules in SDF format were downloaded from PubChem and converted to mol2 format using OpenBabel software. Molecular docking analysis of the protein receptor and the small molecule ligand was then performed using AutoDock software to calculate the binding free energy of the two. The lower the binding energy value, the stronger the binding affinity. Finally, we visualised the docking results using PyMOL software to visually analyse the interaction patterns of the compounds with the target proteins. This experiment provides an important basis for evaluating the potential therapeutic effects of candidate compounds.

### 2.13 Establishment of animal and MCAO models

In this study, we used a middle cerebral artery occlusion (MCAO) model to simulate ischemic stroke to investigate the changes in related gene expression. Since ischemic stroke is mainly a disease of the elderly and the incidence rate increases significantly with age, in order to provide clinical value for future translational medicine, we selected aged (24-27 months) male Wistar rats provided by Beijing Viton Lever Laboratory Animal Co(Compliance with relevant regulations and guidelines and ethical approval from our Animal Ethics Committee). and randomly divided the rats into the MCAO group and the control group of 6 rats each, and the MCAO model was established according to previous reports (25). The rats were then subjected to general anaesthesia with 2% isoflurane. After a midline incision was made, the right common carotid and external carotid arteries were isolated and the internal carotid artery was clamped. The middle cerebral artery was then occluded with nylon sutures to establish the MCAO model for 24 hours. All procedures were performed in strict accordance with the Guide for the Care and Use of Laboratory Animals.

### 2.14 RNA extraction and qPCR

Total RNA was extracted from rat peripheral blood by the TRIzol method and reverse transcribed using the First-strand cDNA Synthesis Mix kit (TaKaRa, Japan) according to the manufacturer’s protocol. Primers used for cDNA amplification are listed in **Supplementary Table 1**. The cDNA was mixed with SYBR Premix Ex Taq2 (TaKaRa, Japan) and synthetic primers for real-time quantitative PCR. PCR conditions were selected according to the manufacturer’s protocol as follows: 2 min at 50°C; 10 min at 95°C; 45 cycles of 10 s at 95°C, 10 s at 60°C and 15 s at 72°C. Relative mRNA expression levels were quantified by normalisation to the expression of the internal reference GAPDH. Gene expression levels are expressed as fold change relative to control.

### 2.15 Statistical analyses

R software (version 4.4.2) was used to examine the data, and the Wilcoxon test was used to compare groups, with *P* < 0.05 defined as a significant difference.

## 3 Results

### 3.1 Identification of differential genes

**Figure 2:**
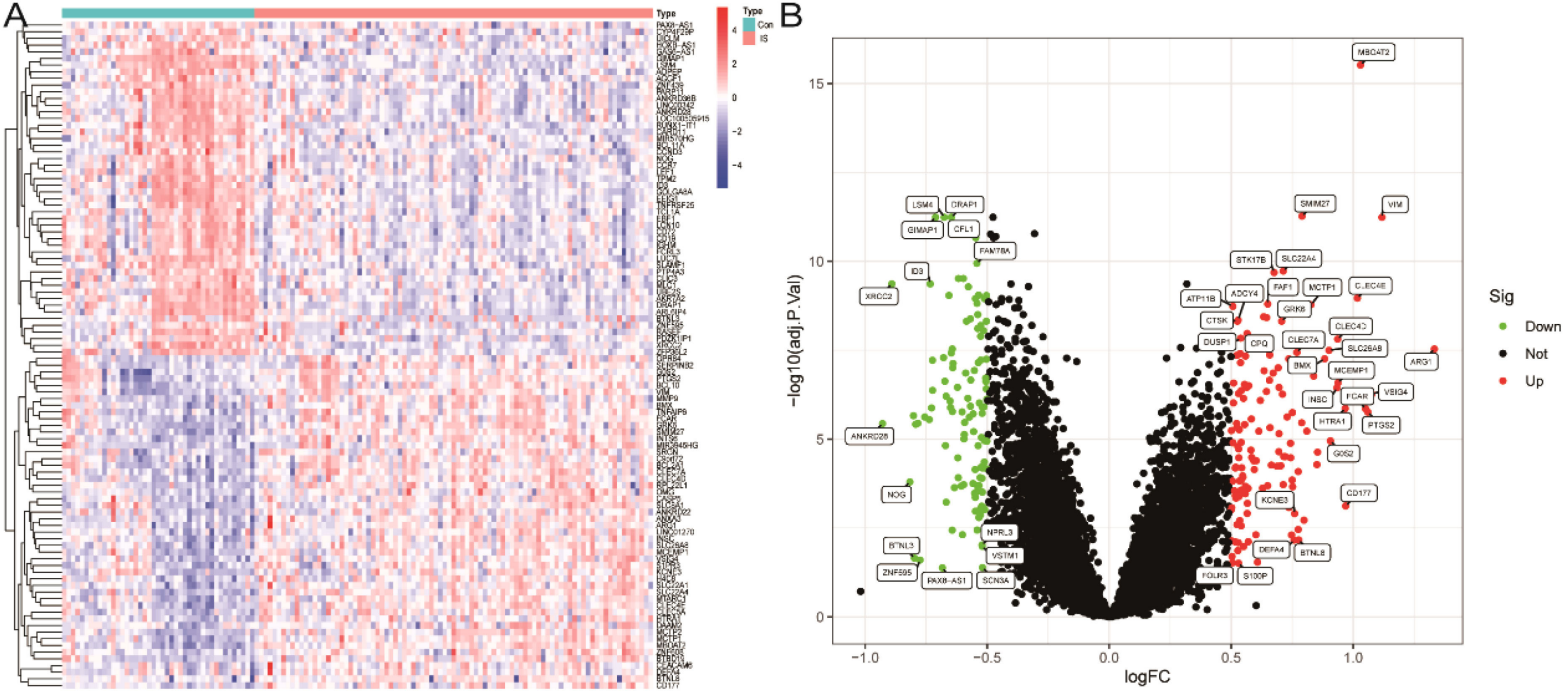
Screening of differentially expressed genes. **(A)** Heatmap of DEGs. **(B)** Volcano plot of DEGs.

We first merged two microarray datasets, GSE58294 and GSE22255, and performed differential analysis after removing batch effects. A total of 243 differentially expressed genes (DEGs) were identified by selecting *p*<0.05 and |log fold change (FC)| > 0.5 as thresholds in the integrated expression matrix and visualised as heat maps and volcano plots (**Figures 2A, B**).

### 3.2 Identification of key modules

**Figure 3:**
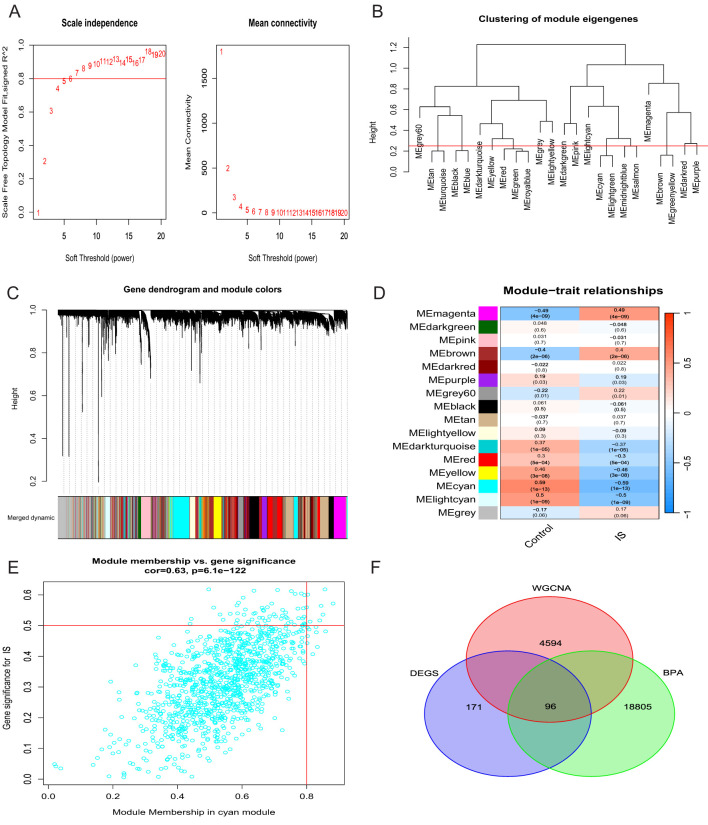
Construction of a weighted gene co-expression network (WGCNA). **(A)** Selection of soft thresholds. **(B)** Dendrogram representing clusters of genes characterising modules. **(C)** Dendrogram of the clustering tree of co-expression modules. **(D)** Heatmap showing the correlation between the 16 modules and the features. **(E)** Genes of the cyan module. **(F)** Venn diagram of overlapping genes.

We selected the merged dataset for weighted gene co-expression network analysis (WGCNA). The constructed scale-free topological network showed the best connectivity when using the PickSoftThreshold function to determine a soft threshold β of 6 (**Figure 3A**). We then set the clustering height to 0.25 for more in-depth analyses and obtained a clustering dendrogram of co-expression modules (**Figure 3C**). Based on the analysis of IS expression profiles, 16 co-expression modules were identified, each represented by a different colour (**Figure 3D**). Correlation analysis of the module features showed that the cyan module had the most significant correlation with IS (cyan; cor = 0.67, P = 0.01) (**Figure 3E**). Therefore, we retained the 1126 genes associated with IS in this module for subsequent studies. Furthermore, by intersection analysis of 243 differentially expressed genes (DEGs) with nucleotide genes and cyan module genes,33 intersecting genes were finally screened (**Figure 3F**).

### 3.3 Gene enrichment analysis

**Figure 4:**
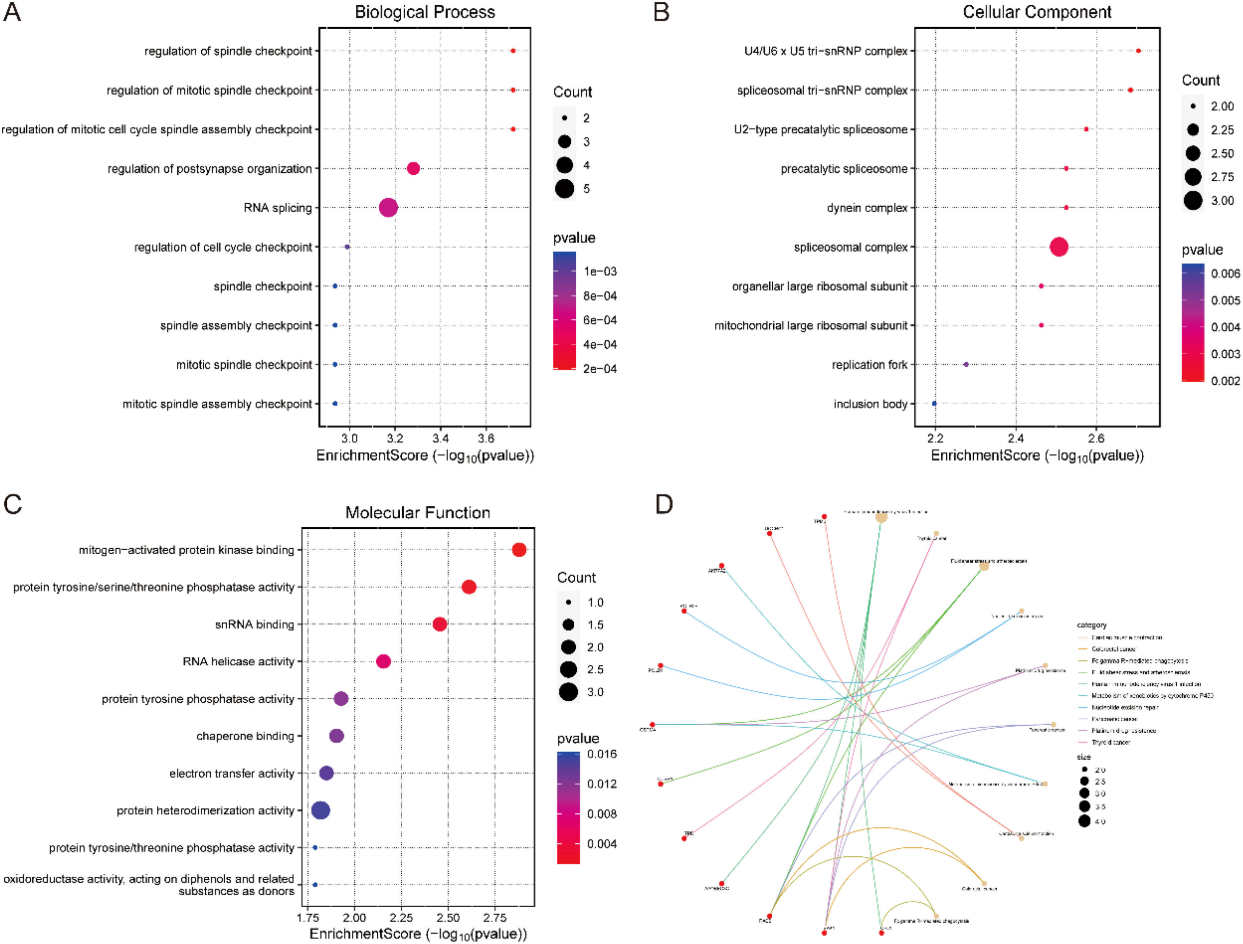
Enrichment analysis of candidate genes. **(A)** BP analysis, **(B)** CC analysis, **(C)** MF analysis. **(D)** KEGG analysis.

By performing gene ontology (GO) and KEGG pathway enrichment analyses on 33 candidate genes, we obtained the following results. In the biological process category analysed by GO, these genes were significantly enriched in processes such as cell cycle regulation, cell division regulation, and regulation of synaptic structure and function (**Figure 4A**). In terms of cellular components, the main features of enrichment included the nucleus, cytoplasm and cytoskeleton (**Figure 4B**). Molecular functional analyses showed significant activities involving enzymatic activity, catalytic activity, and binding activity (**Figure 4C**). KEGG pathway analysis revealed that these candidate genes were enriched in several biologically relevant pathways, including human immunodeficiency virus type 1 infection and nucleotide excision repair-related pathways. Notably, fluid shear stress and atherosclerosis-related pathways were similarly enriched (**Figure 4D**). This suggests that nucleotides may influence the development of IS by modulating a wider range of pathological processes, thereby influencing the development of IS.

### 3.4 Using machine learning to screen for key genes

**Figure 5:**
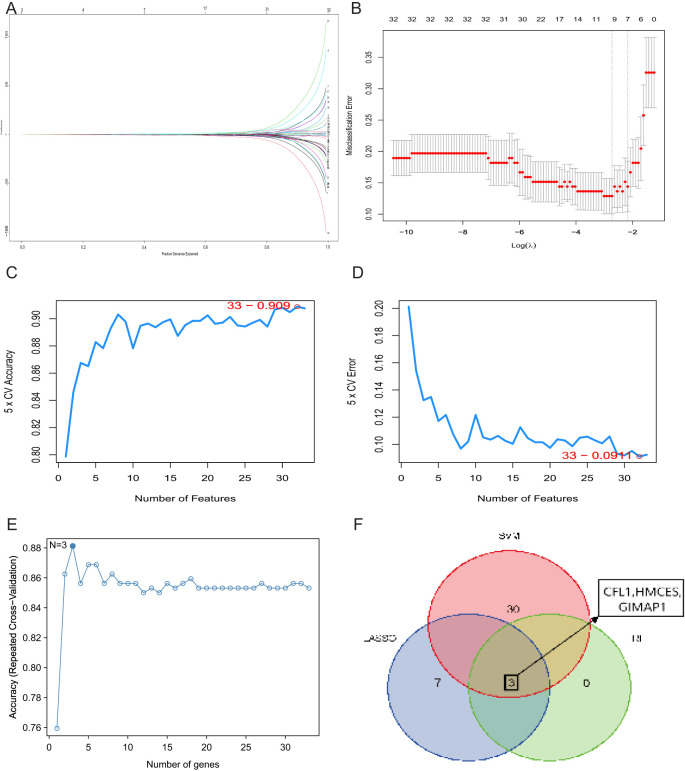
Identification of key genes. **(A)** Cross-validation to select the best-fit parameter logarithm (lambda) in the LASSO regression analysis. **(B)** LASSO coefficient profiles of the candidate genes. **(C)** SVM-RFE analysis selected 33 gene features with an accuracy of 0.909. **(D)** Error of 0.0911. **(E)** RF analysis identified three genes. **(F)** Venn diagram of three key genes shared by LASSO, SVM-RFE and RF algorithms.

To screen for ischemic stroke signature genes, we applied the previously obtained 33 candidate genes to three commonly used feature selection algorithms: SVM-RFE, RF and LASSO. 10 signature genes were obtained from the screening using LASSO regression analysis (**Figures 5A, B**). 33 genetic markers were identified by SVM with an accuracy of 0.909 (**Figures 5C, D**). Subsequently, 33 candidate genes were ranked by importance scores using the random forest method, and three trait genes were screened (**Figure 5E**). Finally, we performed intersection analysis on the results of the three methods and obtained three common key genes (**Figure 5F**).

### 3.5 Assessment of the diagnostic value of the hub genes

**Figure 6:**
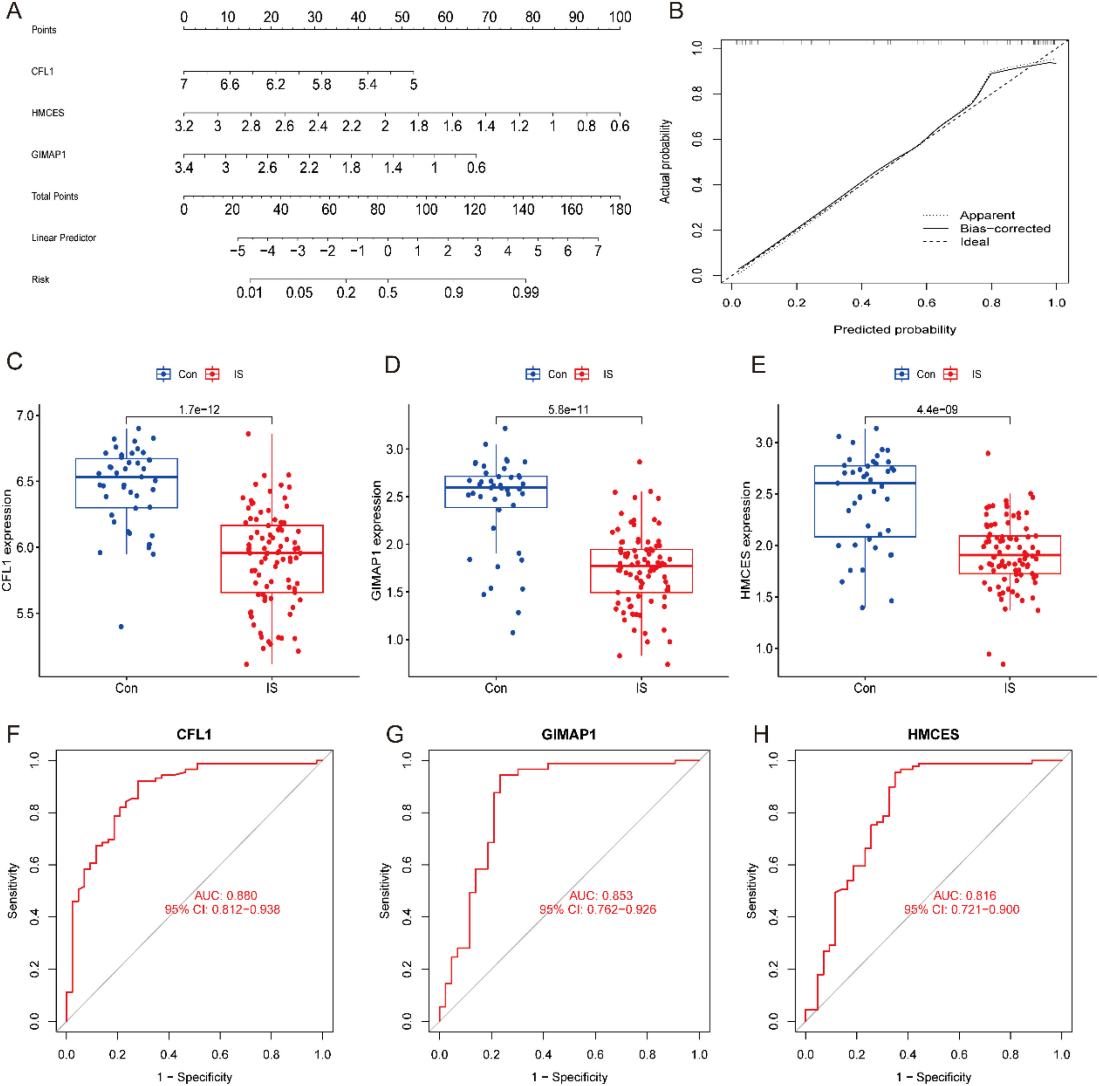
Model construction of column-line diagrams for IS diagnosis and expression of key genes. **(A)** Column graph predicting IS risk. **(B)** Calibration curves assessing the diagnostic potential of the line drawing model. **(C–E)** Expression of key genes in controls and IS. **(F–H)** Diagnostic values of key genes.

A column-line diagram model for IS diagnosis was developed based on *CFL1, HMCES* and *GIMAP1* (**Figure 6A**). The calibration curves showed that the difference between observed and predicted risks was limited, indicating that the column-line graph model performed very well in predicting IS (**Figure 6B**). Analysis by constructing a box plot (Box plot) revealed that the three signature genes were expressed at low levels in the disease group (**Figures 6C–E**). Subsequently, the expression of the three pivotal genes in the screening set and their diagnostic value were further evaluated using ROC curves. The results showed that the AUC values between ischemic stroke (IS) samples and healthy control samples in the screening set were 0.880 (95% CI, 0.812-0.938) for *CFL1* and 0.853 (95% CI, 0.762-0.962) for *GIMAP1*, *HMCES* was 0.816 (95% CI, 0.721-0.900) (**Figures 6F–H**). These results suggest that these three hub genes have good potential in the predictive ability of IS, providing an important basis for their application in clinical diagnosis.

### 3.6 Diagnostic models and TF-mRNA networks

**Figure 7:**
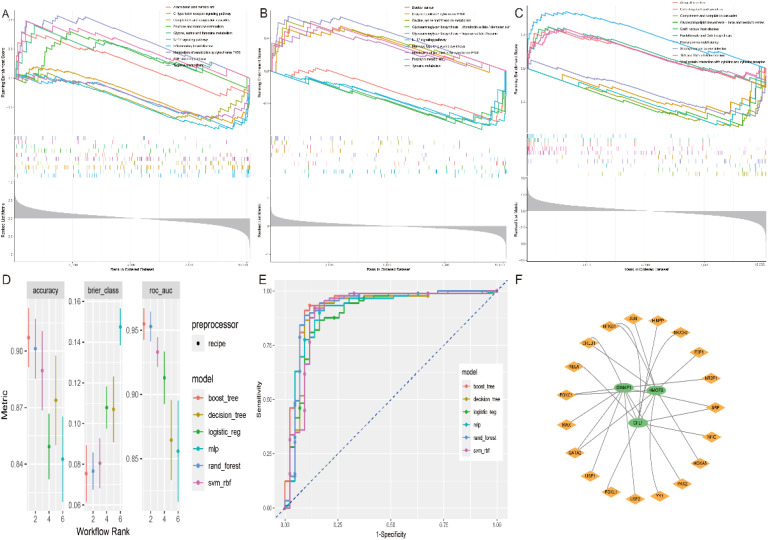
GSEA analysis of key genes, machine learning models and TF-mRNA regulatory networks. GSEA analysis of key genes **(A)** *CFL1*, **(B)** *GIMAP1* and **(C)** *HMCES*. **(D, E)** Accuracy, error and AUC values of the six machine learning models. **(F)** TF-mRNA regulatory network.

Using single-gene GSEA analysis, we found that the pathways enriched in these key genes were mainly involved in immune-related processes such as allograft rejection, primary immunodeficiency, IL-17 signalling, TNF signalling and arachidonic acid metabolism (**Figures 7A–C**). This further supports their key role in ischemic stroke pathogenesis. For machine learning, we used the tidymodels package (v1.1), following the approach recommended by the developers. Each dataset was divided into training and test subsets in an 80:20 ratio. After model development on the training set, the test dataset was only evaluated once. and validated the model in training using k-fold cross-validation with k = 10 and found it to have good predictive power (**Figures 7D–E**). To understand the overall framework of gene regulation and to understand the regulatory relationships. We predicted potential transcription factors for these hub genes using the NetworkAnalyst database. The results showed that the transcription factor NFKB1 regulates both *HMCES* and *CFL1*; CREB1, FOXC1 and GSTA2 regulate *HMCES* and *GIMAP1*; and SRF may regulate both *GIMAP1* and *CFL1*, providing a basis for further elucidation of the gene regulatory network (**Figure 7F**).

### 3.7 Human tissue expression profiles

**Figure 8:**
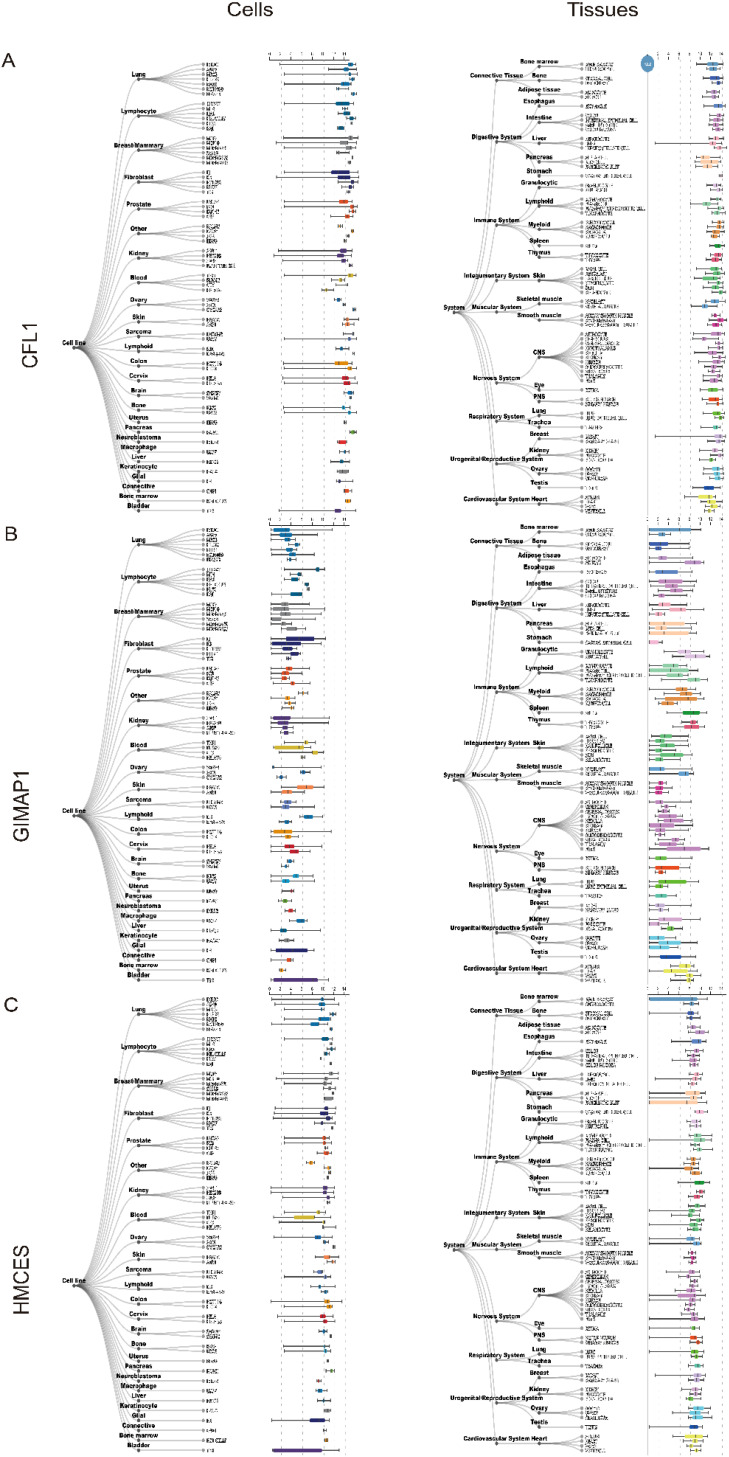
Expression of three key genes, **(A)** *CFL1*, **(B)** *GIMAP1* and **(C)** *HMCES* in human tissues and cells.

Using the Harmonizome database, we analysed the expression of key genes in human tissues (**Figures 8A–C**), with a particular focus on brain and intracerebral cell lines.

### 3.8 scRNA profiling in IS

**Figure 9:**
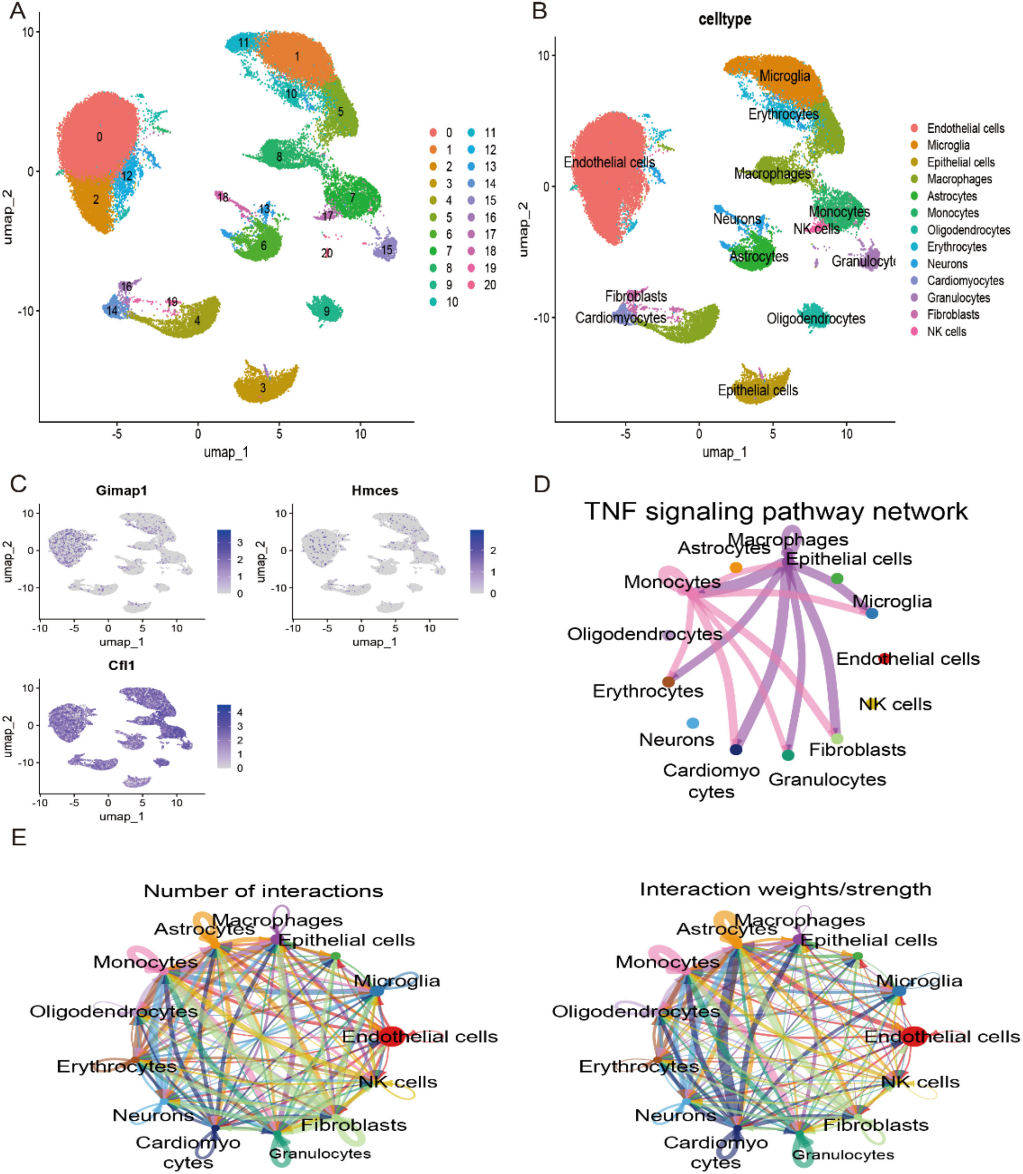
Validation of key genes in the single-cell dataset GSE174574. **(A)** UMAP plot showing the major classifications involved in stroke in the GSE174574 dataset. **(B)** UMAP plot showing the major cell types involved in stroke in the GSE174574 dataset. **(C)** Distribution of key genes across cell types in the GSE174574 dataset. **(D)** TNF signalling pathway network of the GSE174574 stroke immune cell population. **(E)** Crosstalk analysis between GSE174574 stroke immune cells.

To investigate the distribution of the three key genes in various cell types in stroke samples, we classified and cellularly annotated the single-cell samples we obtained. We identified 21 clusters in the GSE174574 dataset with 13 different cell types: endothelial cells, microglia, macrophages, astrocytes, monocytes, oligodendrocytes, erythrocytes, neuronal cells, cardiomyocytes, granulocytes, fibroblasts, and NK cells (**Figures 9A, B**). We observed a major distribution of hub genes in these cells in the GSE174574 stroke samples (**Figure 9C**). Next, we analysed intercellular communication between different types of immune cells in the stroke setting using CellChat (**Figure 9E**). Since the occurrence of stroke is associated with a variety of pathological processes such as inflammatory response, immune regulation and cell death. The communication network of TNF signalling pathway in immune cells was mainly examined in stroke samples (**Figure 9D**).

### 3.9 Correlation of key genes with immune infiltrating cells

**Figure 10:**
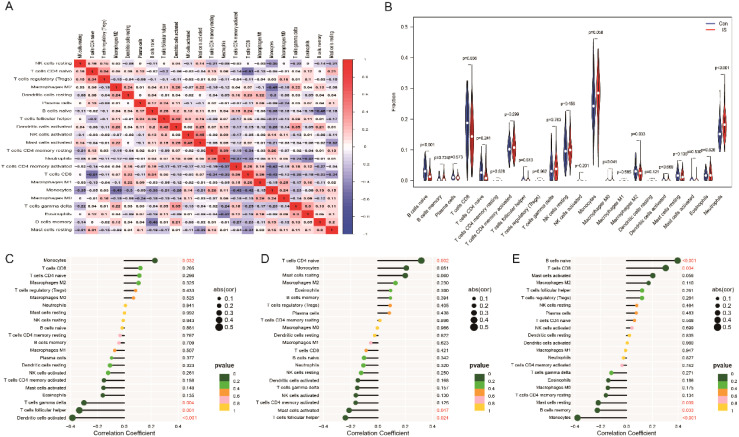
Distribution of immune cells in IS. **(A)** Heatmap of immune cell expression in IS. **(B)** Differences in immune cells between control and IS. **(C-E)** Correlation analysis of *CFL1, GIMAP1* and *HMCES* with immune cells.

We analysed immune cell infiltration in the ischemic stroke (IS) and normal control groups using the CIBERSORT algorithm (**Figure 10A**). The results showed that the abundance of naive B cells and CD8+ T cells was significantly lower in the IS group compared to the normal group, whereas the abundance of monocytes and neutrophils was significantly higher (**Figure 10B**). Further analysis revealed that the expression levels of these three hub genes (*CFL1, GIMAP1* and *HMCES*) correlated with the abundance of most immune cells (**Figures 10C–E**). It is suggested that these key genes may regulate the function of immune cells and influence the development of ischemic stroke.

### 3.10 Drug prediction and molecular docking for IS therapy

**Figure 11:**
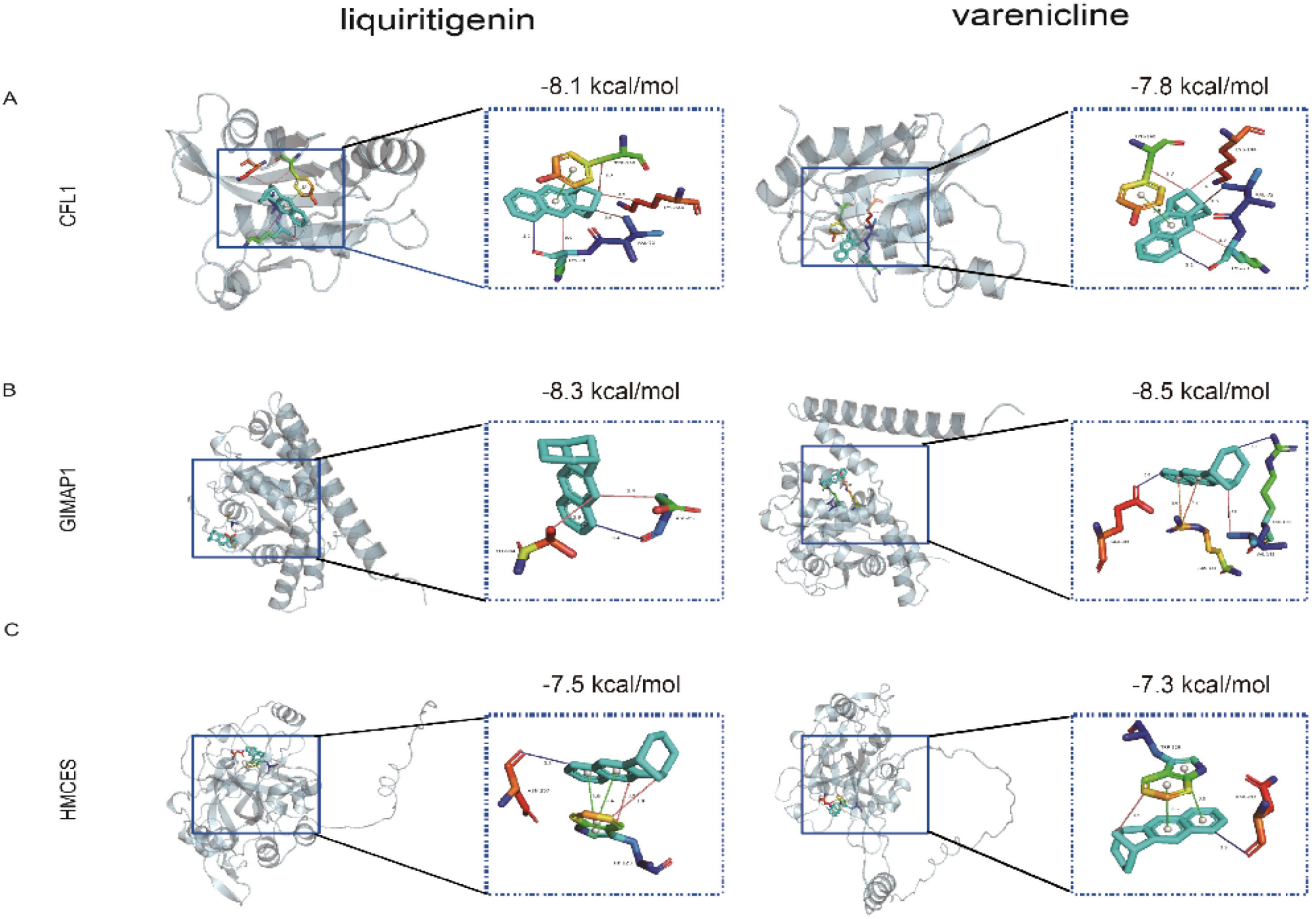
Molecular docking patterns of liquiritigenin or varenicline with three target proteins. Docking results of **(A)** CFL1, **(B)** GIMAP1, **(C)** HMCES with liquiritigenin and varenicline. LYS, lysine; VAL, valine; TYR, Tyrosinase; ASP, aspartic acid; THR, L-Threonine; GLU, glutamate; ARG, arginine; ASN, asparticacid; TRP, L-tryptophan.

In summary, this study successfully predicted potential therapeutic small molecule compounds (all connectivity scores >0.7) by submitting the differentially expressed genes to the Connectivity Map (Cmap) database (**Supplementary Figure 1**), and we selected the two drugs with the largest positive and negative scores. Molecular docking analysis showed that these three hub genes formed good binding conformations with both liquiritigenin and varenicline. The most stable binding conformation of two compounds to each target protein was also visualised using Pymol software, which included multiple intermolecular interactions such as hydrogen bonding, hydrophobic forces, π-cation interactions and π-stacking (**Figure 11**), suggesting that they could be potential targets for these two compounds.

### 3.11 Hub gene validation

**Figure 12:**
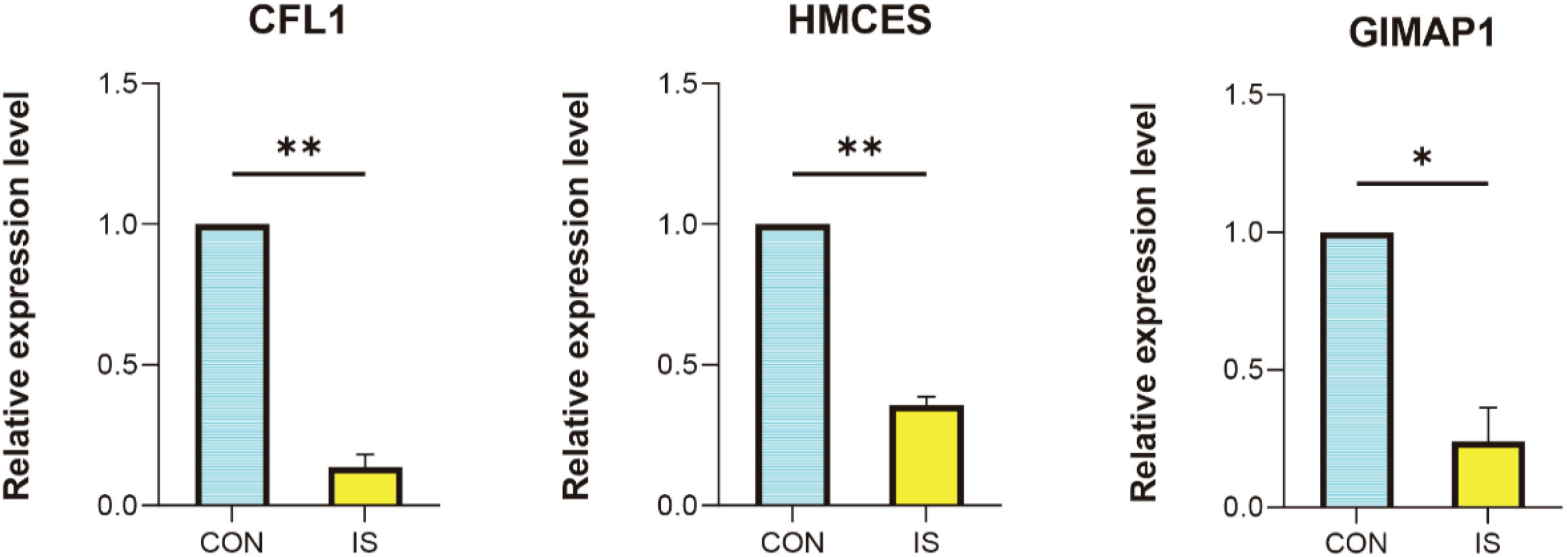
qPCR validation. Comparison of gene expression between IS rat model and control in *CFL1*, *HMCES* and *GIMAP1*. *P < 0.05, **P < 0.01 vs. CON.

We successfully established a rat MACO (middle artery permanent occlusion) ischemic stroke model. To further investigate the expression changes of key genes in this model, peripheral blood was collected from MACO and control rats, and RNA was extracted and analysed by qPCR. The results showed that all three genes, *GIMAP1, HMCES and CFL1*, were significantly downregulated in IS compared to the control group (**Figure 12**). The changes in the expression of these genes provide important clues for further exploration of the pathogenesis and therapeutic targets of ischemic stroke, and lay the foundation for subsequent translational medicine research.

## 4 Discussion

Globally, stroke has become a serious public health problem, affecting more than 795,000 people and killing about 140,000, or 1 in 20, each year, making it the fifth leading cause of death worldwide (26). At the same time, the total cost of stroke in the United States, including healthcare services, medication costs and work loss due to stroke, is approximately $34 billion annually (27). Because stroke is a complex process involving multiple factors, current treatments for IS still have a poor prognosis due to the narrow therapeutic window, potential risk of bleeding and subsequent reperfusion injury, etc (28). In contrast, Nucleotide metabolism plays a key role in the onset and development of stroke and is an important factor in understanding stroke mechanisms and identifying new therapeutic targets. Abnormalities in nucleotide metabolism can lead to inadequate cellular energy supply, dysregulated inflammatory responses and reduced cellular repair capacity, thereby exacerbating brain tissue damage.First,The ischaemic state following a stroke leads to a lack of energy supply to brain cells and a significant decrease in ATP levels (29). Increased ATP synthesis, support for cell survival and improved energy metabolism can be achieved by regulating nucleotide metabolism, which is essential for restoring brain cell function (30). In addition, ischemic stroke is associated with a pronounced inflammatory response and nucleotide metabolites (e.g. adenosine) play an important role in regulating inflammation, inhibiting the release of pro-inflammatory cytokines and reducing brain damage (31). Effective modulation of this pathway could provide neuroprotection for stroke patients. In addition, nucleotides are essential for DNA and RNA synthesis and promote the proliferation and differentiation of neural stem cells, thereby supporting the regeneration of brain tissue after stroke and significantly improving functional recovery (32). Therefore, it is extremely important to search for stroke biomarkers related to nucleotide metabolism. This will not only help in the early diagnosis and assessment of stroke severity, but may also provide an important basis for the development of new therapeutic strategies. Our study aims to explore the potential biomarkers and therapeutic targets of nucleotide metabolism in stroke. This will open up new avenues for the prevention and treatment of stroke and enable the development of more targeted interventions to reduce stroke morbidity and mortality and ultimately improve patients’ quality of life.

In this study, we investigated the molecular mechanisms of ischemic stroke (IS) using an integrated bioinformatics approach. First, we screened genes related to nucleotide metabolism from the Genecards database and retrieved and analysed two related datasets from the GEO database. Through merging and differential analysis, we identified 243 significantly differentially expressed genes. Using weighted gene co-expression network analysis (WGCNA) and three machine learning methods (LASSO, support vector machine SVM and random forest RF), we identified three core immune-related diagnostic biomarkers: *CFL1, HMCES* and *GIMAP1*. The role of these core immune genes in IS was validated by enrichment analysis, which showed that they were significantly enriched in several immune-related processes, particularly nucleotide excision repair and the fluid shear stress pathway associated with atherosclerosis. In addition, we comprehensively analysed the infiltration levels of 22 immune cell types using the CIBERSORT algorithm. Compared with controls, the expression of naive B cells and CD8+ T cells was downregulated in IS patients, whereas the expression of monocytes and neutrophils was significantly increased.The expression levels of *CFL1, HMCES* and *GIMAP1* correlated with the infiltration levels of a wide range of immune cell types, further confirming their important role in IS.

To further validate these findings, we used single-cell transcriptome sequencing data to investigate the expression patterns of these genes in different cell types and analysed the cellular communication networks between these cell types using CellChat. These analyses revealed the interaction of multiple immune cells in stroke pathogenesis through the TNF pathway. For example, early studies demonstrated the regulation of TNF expression in microglia and its role in stroke mechanisms (33) and the role of TNF-α in exacerbating ischaemic conditions after stroke (34). In addition, microglia/macrophages deficient in vitamin D receptors exhibit a pro-inflammatory phenotype characterised by significant secretion of TNF-α, which is strongly associated with poor stroke outcome (35). Taken together, the three genes *CFL1, HMCES* and *GIMAP1* may not only serve as diagnostic biomarkers for stroke, but also play a key role in the pathophysiological process of stroke. By revealing the regulatory role of the TNF pathway in the pathogenesis of stroke, our study provides an important scientific basis for exploring new therapeutic targets.

To further understand the interaction between nucleotide metabolism and immune infiltration, we hypothesised that these key genes may influence the pathological process of ischemic stroke through multiple mechanisms. First, nucleotide metabolites (e.g., ATP and adenosine) play an important role in immune regulation. ATP, as a key effector molecule of damage-associated molecular patterns (DAMPs), mediates neutrophil chemotactic migration via the P2Y2 receptor (36) and exacerbates acute inflammatory responses by activating the NLRP3-inflammasome-IL-1β signalling axis (37). In contrast, adenosine inhibits NF-κB nuclear translocation through an A2A receptor-dependent pathway, significantly downregulates the expression of pro-inflammatory factors such as TNF-α and IL-6, and blocks neutrophil infiltration (38), forming a metabolism-dependent negative feedback regulatory mechanism for inflammation. Second, activated immune cells exhibit a significant metabolic phenotypic switch from oxidative phosphorylation to aerobic glycolysis and nucleotide *de novo* synthesis pathways. This metabolic remodelling not only meets the bioenergetic demands of rapid proliferation, but the metabolic intermediates (e.g. adenosine) also form a metabolic-immunoregulatory loop via the autocrine/paracrine pathway. In particular, the *HMCES*-mediated DNA damage repair pathway may influence the time course and intensity of inflammatory responses by regulating genomic stability (39), whereas *CFL1*-dependent cytoskeletal remodelling directly regulates the tissue infiltration capacity of immune cells (40). These findings reveal a complex network of interactions between metabolic regulation and immune response, and provide a theoretical basis for the development of small molecule intervention strategies (e.g. purine analogues, adenosine receptor modulators) targeting nucleotide pathways.

The CMap database predicts highly relevant molecular drugs for the treatment of IS (linkage score > 0.7). Liquiritigenin is a flavonoid extracted from liquorice, which has been shown to have a variety of biological activities, including anti-inflammatory and antioxidant activities; it is able to inhibit the inflammatory cascade induced by cerebral ischaemia/reperfusion (41), such as reducing the expression of cytokines, chemokines and inflammatory enzymes. Varenicline is a partial agonist of the nicotinic acid receptor, which can increase the excitability and activity of neurons and improve neuronal function. Varenicline has some antioxidant activity, which may scavenge free radicals and reduce damage to neurons caused by oxidative stress (42, 43). Further research is needed to analyse the effects of these molecules on behavioural tests in IS patients.

Currently, the diagnosis of IS relies heavily on neuroimaging. An accurate diagnosis of IS and early preventive measures are essential to alleviate suffering and improve the prognosis of the disease. Based on a bioinformatics approach, this is the first study to identify three key genes involved in nucleotide metabolism (*CFL1, HMCES* and *GIMAP1*) that are closely associated with IS. CFL1 is an ATP-dependent cytoskeletal regulatory protein that is mainly involved in the dynamic reorganisation of the cytoskeleton (44). Its activity is directly influenced by nucleotide metabolism, in particular ATP availability, and plays a key role in neuronal development, synaptic function and synaptic plasticity (40). Its abnormal expression and function have been implicated in neurodegenerative diseases such as Alzheimer’s disease and Parkinson’s disease. In Alzheimer’s disease, CFL1 is enriched in pathological protein aggregates, leading to disruption of the neuronal cytoskeletal structure and impairing neuronal function and survival (45). In Parkinson’s disease, overactivation leads to an imbalance in F-actin dynamics, triggering neuronal synaptic dysfunction and neuronal death (46). HMCES is a DNA repair protein involved in the maintenance of genome integrity and plays a key role in the maintenance of genome integrity (47). Dysregulation of nucleotide metabolism can affect the function of HMCES, leading to the accumulation of DNA damage and increasing cellular susceptibility to damage (48). Impaired repair of DNA damage leads to genomic instability, which is particularly evident in neurodevelopmental and neurodegenerative diseases. For example, patients with Alzheimer’s disease often have functional defects in DNA damage and repair mechanisms that are closely associated with reduced HMCES activity (49). GIMAP1 is a GTPase that regulates immune cell activity and is involved in cell proliferation and apoptosis (50, 51). Its function is closely linked to nucleotide metabolism, as activation of the immune response requires sufficient energy and nucleotide support (52). Abnormal metabolism can lead to dysregulation of GIMAP1 function, which in turn triggers abnormal immune responses. This immune dysregulation may lead to chronic inflammation in the central nervous system, which has been shown to be closely associated with the development of several neurological diseases, including multiple sclerosis and Alzheimer’s disease (53). Taken together, these three genes and their encoded proteins play important roles in the development of neuronal cell injury, neuroinflammatory response and neurological dysfunction. They provide new research ideas and potential therapeutic targets for the prevention and treatment of post-stroke sequelae.

However, the study has some limitations. First, the model approach may not be able to fully mimic IS by identifying the 3 hub genes by qPCR and validating their localisation and distribution; second, the scope of this study was insufficient to include detailed *in vivo* and *in vitro* validation. Third, the study should have included additional clinical and demographic characteristics of the patients for further subgroup analyses. Addressing these limitations in future studies will improve the reliability and translational potential of our findings.

## 5 Conclusion

By integrating machine learning and transcriptomic approaches, our study provides an in-depth analysis of nucleotide metabolism-related gene profiles in ischemic stroke patients and identifies three potential key regulatory genes that are closely related to immune system activity. This provides a new perspective for understanding the complex pathogenesis of ischemic stroke, lays the foundation for future drug development based on key targets, and has important clinical translational value.

## Data Availability

The original contributions presented in the study are included in the article/**Supplementary Material**. Further inquiries can be directed to the corresponding authors.
