# Supplementary material for: Integrating machine learning and multi-omics analysis to reveal nucleotide metabolism-related immune genes and their functional validation in ischemic stroke
Source: Front Immunol. 2025 Mar 26;16:1561544. doi: 10.3389/fimmu.2025.1561544 (PMC11979214; doi:10.3389/fimmu.2025.1561544)

**Supplementary Information:**

The online version contains supplementary material available at:

Additional file 1.

Table S1. Primer sequences used in this study.

| **Genes** |  | **Primers (5′–3′)** |
| --- | --- | --- |
| Gimap1 | F | GGGCCACATCAGAGACTTTCA |
|  | R | CTGGAGTCATCCTCTGAGCCATA |
| Hmces | F | AGCTCTGAAGCTAATCCACCC |
|  | R | CTTGCCTTGGGCTCCTTCTTA |
| Cfl1 | F | TTCCGGAAACATGGCCTCTG |
|  | R | CACTGCCTTCTTGCGTTTCT |
| GAPDH | F | GAGTCAACGGATTTGGTCGT |
|  | R | GACAAGCTTCCCGTTCTCAG |

Additional file 2.

Figure S1. Drug prediction. CMap instances organised by compound represent the most significant positive and negative correlations with effect on IS.


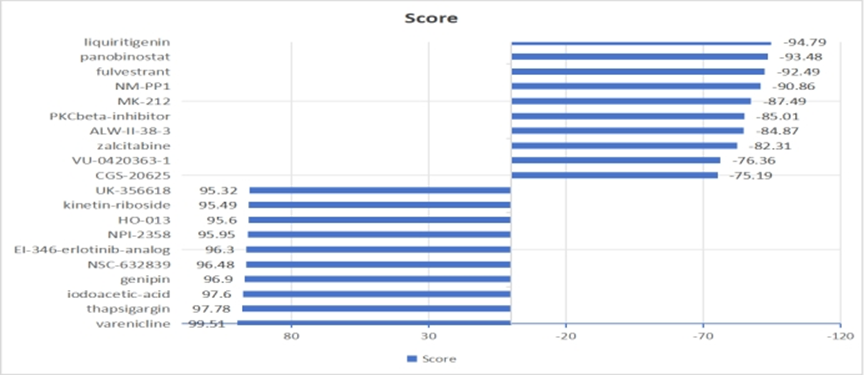

Supplement: Supplementary file 1 [file Table1.doc]
